# Effects of Cyclic High Ambient Temperature and Dietary Supplementation of Orotic Acid, a Pyrimidine Precursor, on Plasma and Muscle Metabolites in Broiler Chickens

**DOI:** 10.3390/metabo10050189

**Published:** 2020-05-12

**Authors:** Saki Shimamoto, Kiriko Nakamura, Shozo Tomonaga, Satoru Furukawa, Akira Ohtsuka, Daichi Ijiri

**Affiliations:** 1 Department of Agricultural Sciences and Natural Resources, Kagoshima University, Korimoto, Kagoshima 890-0065, Japan; shimamoto@agr.niigata-u.ac.jp (S.S.); k5023091@kadai.jp (K.N.); ohtsuka@chem.agri.kagoshima-u.ac.jp (A.O.); 2 Graduate School of Science and Technology, Niigata University, Nishi-ku, Niigata 950-2181, Japan; 3 Division of Applied Biosciences, Graduate School of Agriculture, Kyoto University, Sakyo-ku, Kyoto 606-8502, Japan; tomonaga.shozo.4n@kyoto-u.ac.jp; 4 Furukawa Research Office Co. Ltd., Setagaya-ku, Tokyo 157-0066, Japan; furukawa@furukawa-res.co.jp

**Keywords:** chickens, heat stress, lipid peroxidation, metabolomics, orotic acid

## Abstract

The aim of this study was to evaluate the effects of high ambient temperature (HT) and orotic acid supplementation on the plasma and muscle metabolomic profiles in broiler chickens. Thirty-two 14-day-old broiler chickens were divided into four treatment groups that were fed diets with or without 0.7% orotic acid under thermoneutral (25 ± 1 °C) or cyclic HT (35 ± 1 °C for 8 h/day) conditions for 2 weeks. The chickens exposed to HT had higher plasma malondialdehyde concentrations, suggesting an increase in lipid peroxidation, which is alleviated by orotic acid supplementation. The HT environment also affected the serine, glutamine, and tyrosine plasma concentrations, while orotic acid supplementation affected the aspartic acid, glutamic acid, and tyrosine plasma concentrations. Untargeted gas chromatography–triple quadrupole mass spectrometry (GC-MS/MS)-based metabolomics analysis identified that the HT affected the plasma levels of metabolites involved in purine metabolism, ammonia recycling, pyrimidine metabolism, homocysteine degradation, glutamate metabolism, urea cycle, β-alanine metabolism, glycine and serine metabolism, and aspartate metabolism, while orotic acid supplementation affected metabolites involved in pyrimidine metabolism, β-alanine metabolism, the malate–aspartate shuttle, and aspartate metabolism. Our results suggest that cyclic HT affects various metabolic processes in broiler chickens, and that orotic acid supplementation ameliorates HT-induced increases in lipid peroxidation.

## 1. Introduction

Ambient temperatures above the thermoneutral zone cause environmental heat stress. Chickens are more vulnerable to heat stress than other domestic animals, because they lack sweat glands and have higher body temperatures [1,2]. Under such high ambient temperature (HT) conditions, the generation of reactive oxygen species increases in various body tissues as the heat load increases [3]. This results in a range of physiological changes that can severely depress growth performance and meat yield [4,5] and reduce meat quality, accompanied by increasing lipid peroxidation levels [6,7] and drip loss [8,9], decreasing the share force value [10,11] and changing meat color [12,13,14].

Gas chromatography–mass spectrometry (GC-MS)-based, untargeted metabolomics analysis has been used to comprehensively characterize the effects of HT on the physiology of chickens [15], and has found that the plasma levels of 38 metabolites changed significantly when chickens were exposed to chronic heat (38 °C) for 4 days. These altered metabolites indicated that such heat exposure affected 35 metabolic processes, including the sulfur amino acid metabolic pathway, the kynurenine pathway in tryptophan metabolism, and nucleic acid metabolism [15]. In agreement with this observation, dietary supplementation with either a sulfur amino acid (methionine) or tryptophan, which is mainly metabolized to kynurenine, has been reported to alleviate the negative effects of HT [16,17]. However, the involvement of nucleic acids and their metabolites in the metabolic changes that occur in chickens kept in HT environments remains unclear.

Orotic acid, which is found in high concentrations in cow’s milk, is a key intermediate in the pyrimidine biosynthesis pathway [18]. Pyrimidine nucleotides are important constituents of RNA and of the phospholipids present in cell membranes. Orotic acid can enter the de novo synthesis pathway for pyrimidines beyond the rate-limiting step, and thereby improve throughput. In chickens, the plasma level of orotic acid was found to significantly decrease, by approximately 60%, in response to short-term chronic heat exposure [15], suggesting that the plasma orotic acid concentration may be important under HT conditions.

In this study, we evaluated the effects of prolonged heat exposure and feeding orotic acid at 0.7% of the diet on the growth performance, plasma and muscle lipid peroxidation levels, and plasma and muscle metabolite concentrations of broiler chickens. To mimic realistic HT conditions, a cyclic HT environment (35 ± 1 °C for 8 h/day) was used. In addition, a food-grade orotic acid (Lactoserum; Matsumoto Trading Co., Ltd., Tokyo, Japan), which contains more than 98% of orotic acid monohydrate, was used for dietary supplementation.

## 2. Results

The final body weight, body weight gain, and feed conversion ratio were not affected by either the HT or orotic acid supplementation, whereas the feed intake was significantly depressed in the chickens kept under the HT conditions (Table 1). In addition, the body temperatures of the chickens kept in the HT environment were significantly increased compared with those of the chickens kept under thermoneutral conditions. However, orotic acid supplementation alleviated the increase in body temperature under the HT conditions.

Although the weights of the leg muscles were not affected by the rearing temperature, the weights of the breast muscles, breast tender muscles, livers, and hearts were lower in the chickens kept under the HT conditions (Table 2). The yield of the breast muscles (the ratio of breast muscles weight to body weight) was the highest in the chickens supplemented with orotic acid and kept in the thermoneutral environment compared to the other three groups of chickens (Table S1). However, the yield of neither the breast tender muscles nor the leg muscles were different among these four groups. Furthermore, the weight of the abdominal fat tissue was increased by the HT environment.

**Figure 1:**
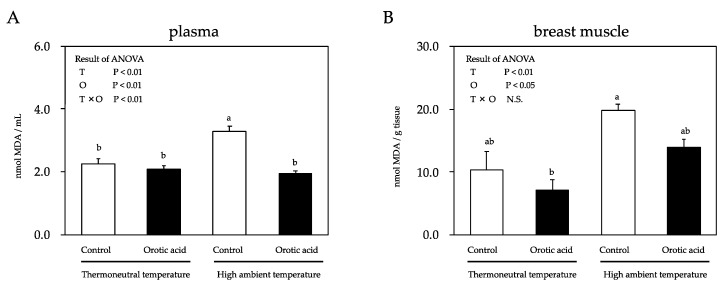
Effects of a cyclic high ambient temperature and feeding orotic acid on the plasma (**A**) and muscle (**B**) malondialdehyde (MDA) concentrations of broiler chickens. Results are expressed as mean ± standard error of the mean (SEM) (*n* = 8). Means with the same superscript letter within columns are not significantly different at *p* < 0.05. T: the effect of high ambient temperature; O: the effect of feeding orotic acid; T × O: the statistical interaction between high ambient temperature and feeding orotic acid.

The malondialdehyde (MDA) concentration, which serves as an index for the lipid peroxidation level, was significantly affected by either temperature or orotic acid supplementation. The plasma MDA concentration of chickens fed a control diet and kept under the HT condition was significantly increased compared with the chickens kept in the thermoneutral environment (Figure 1). However, under the HT condition, the dietary supplementation of orotic acid decreased the plasma MDA concentration compared to the control diet. On the other hand, the HT condition did not significantly increase the muscle MDA concentration of chickens fed a control diet. Furthermore, dietary supplementation of orotic acid did not alleviate that of chickens under the HT condition.

Table 3 shows the plasma-free amino acid concentrations of the chickens kept under thermoneutral or HT conditions. There were significant effects of ambient temperature on the plasma serine, glutamine, and tyrosine concentrations, while orotic acid supplementation significantly affected the plasma aspartic acid, glutamic acid, and tyrosine concentrations. The two-way ANOVA revealed no significant interaction between the HT and orotic acid supplementation on these 18 plasma-free amino acids.

Untargeted gas chromatography–triple quadrupole mass spectrometry (GC-MS/MS)-based metabolomics analysis identified a total of 172 metabolites in the plasma of the chickens (Table 4 and Table S2). Of these metabolites, 23 were significantly affected by HT, of which 11 were significantly increased and 12 were decreased (Table 4). On the other hand, 12 were affected by orotic acid supplementation, of which 11 were significantly increased, and 1 was decreased. However, the two-way ANOVA revealed no significant interaction between the temperature and dietary treatments for the plasma metabolites.

The enrichment analysis indicated that nine and four metabolic pathways were affected by the HT environment and the orotic acid supplementation, respectively (Table 5).

The free amino acid and carnosine concentrations in the broiler chickens’ breast muscles are shown in Table 6. The HT treatment significantly affected the serine, glutamine, arginine, methionine, and phenylalanine concentrations, while the orotic acid supplementation significantly affected the histidine, methionine, and carnosine concentrations. The two-way ANOVA revealed no significant interaction between the temperature and dietary treatments for the concentrations of the 18 free amino acids and carnosine in the muscle.

## 3. Discussion

Reactive oxygen species (ROS), such as singlet oxygen, hydrogen peroxide, and hydroxyl radicals, are highly reactive molecules produced by mitochondria [19]. Under high ambient temperature conditions, ROS generation increases in various body tissues of chickens as the heat load elevates [3], and consequently oxidizes and impairs lipids, proteins, and DNA [20]. Because chicken muscle has a high polyunsaturated fatty acid content, making it more sensitive to oxidative deterioration [21], the oxidation of such lipids negatively influences their industrial values (i.e., growth performance, meat yield, and meat quality) [4,5,6,7,8,9,10,11,12,13,14].

Under HT conditions, chickens have been known to show lower growth rate and feed efficiency, accompanied by decreasing meat yield [4,5]. In this study, although the HT environment did not affect the final body weight, body weight gain, or feed conversion ratio of the broiler chickens, it significantly depressed their feed intake and increased their average body temperature. These results concur with those of a previous study, which examined the effects of a similar HT environment [13]. In addition, the HT environment affected the weights of the breast muscles, breast tender muscles, livers, hearts, and abdominal fat tissue. In particular, the yield of breast muscle was significantly decreased by the HT environment. Furthermore, the plasma lipid peroxidation level was higher in the chickens kept under the HT conditions than those kept in the thermoneutral environment. Therefore, these results suggest that the HT environment used in this study could realistically induce the negative effects commonly observed in broiler chickens kept in heat stress-inducing environments.

In this study, most of the plasma amino acid concentrations were unaffected by HT, with the exception of the serine, tyrosine, and glutamine concentrations. This was particularly notable in the chickens fed the basal diet, in which the HT environment significantly decreased the plasma glutamine concentration. This concurred with the results of a previous study, which reported that chickens kept under HT conditions had lower plasma glutamine concentrations, in addition to poorer performance and carcass characteristics [22]. Although glutamine is a non-essential amino acid, it has been reported to promote enterocyte proliferation and survival, and regulate intestinal barrier function under a variety of stress conditions [23]. Furthermore, intestinal morphology and permeability were found to be disrupted under HT conditions, with this being accompanied by an increase in the plasma endotoxin concentration in broiler chickens [24]. These results suggest that glutamine is utilized to maintain intestinal integrity in broiler chickens under heat-stress conditions.

**Figure 2:**
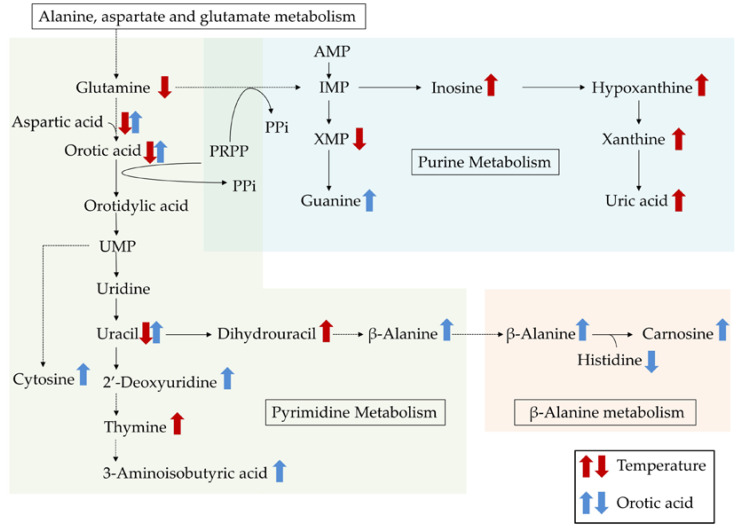
Integrated overview of the metabolic changes induced by either a cyclic high ambient temperature or by feeding orotic acid. Red arrows indicate high ambient temperature-induced changes in the metabolites, and blue arrows indicate orotic acid-induced changes. The metabolic scheme was based on information gathered from the KEGG PATHWAY Database (http://www.genome.jp/kegg/pathway.html).

In addition, glutamine is known to be a precursor for the synthesis of purine and pyrimidine nucleotides, which are essential for DNA synthesis and the proliferation of cells [25]. In rats, GC-MS and liquid chromatography–mass spectrometry-based metabolomics analysis of the plasma indicated that short-term chronic heat exposure (37 °C for 48 h) altered pyrimidine and purine degradation [26]. To investigate the effects of an HT environment on metabolite profiles in the plasma of chickens, we performed untargeted GC-MS/MS-based metabolomics analysis. This analysis identified 172 metabolites in the plasma, of which 23 were significantly affected by the HT environment. The HT environment particularly affected the plasma levels of purine- (inosine, hypoxanthine, xanthine, xanthosine monophosphate, and uric acid) and pyrimidine-related (uracil, dihydrouracil, and thymine) metabolites. The changes in these metabolites are illustrated in Figure 2, with a general increase in concentration in response to the HT environment being apparent.

One possible explanation for the changes in the metabolite concentrations observed may be the higher oxidative stress levels in the broiler chickens kept under the HT conditions. Uric acid is known to act as an antioxidant in the plasma [27], and is thought to stabilize ascorbic acid [28]. However, the concentration of ascorbic acid was lower in the plasma of the chickens kept in the HT environment than in the chickens kept under thermoneutral conditions. Another possibility is that these changes were linked the higher energy expenditure of the broiler chickens kept at HT. When broiler chickens are exposed to environmental temperatures above 31 °C, they increase heat production [29], suggesting an increase in adenosine triphosphate (ATP) production. Adenylate kinase, which acts as a regulator for phosphate nucleotide levels inside cells, converts two adenosine diphosphate (ADP) molecules to one ATP molecule and one adenosine monophosphate (AMP) molecule [30]. Since the concentration of AMP is maintained at a much lower level than that of either ADP or ATP [31], the generated AMP may be catabolized to form hypoxanthine, xanthine, or uric acid.

The enrichment analysis identified that the HT environment induced changes in purine metabolism, ammonia recycling, pyrimidine metabolism, homocysteine degradation, glutamate metabolism, the urea cycle, β-alanine metabolism, glycine and serine metabolism, and aspartate metabolism. Seven of these pathways (excluding homocysteine degradation and aspartate metabolism) concur with the findings of a previous study on the effects of short-term chronic heat exposure on the plasma metabolomic profiles of chicks [15]. Furthermore, the plasma homocysteine concentration is considered an oxidative stress marker [32]. In Japanese quail, HT increased both the homocysteine concentration and the lipid peroxidation level in the plasma [33,34], and it has been suggested that chickens kept under HT conditions have higher plasma homocysteine concentrations. Furthermore, the untargeted GC-MS/MS-based metabolomics analysis indicated that the HT environment decreased the plasma level of aspartic acid, which is a precursor for the synthesis of pyrimidines. These results suggest that although purine and pyrimidine synthesis pathways in chickens were enhanced in response to the HT conditions, the levels of related metabolites (e.g., glutamine, aspartic acid, and ascorbic acid) may have been disrupted.

It is noteworthy that the HT environment changed not only pyrimidine metabolism, but also aspartate metabolism and β-alanine metabolism, because both pyrimidine metabolism and aspartate metabolism are closely related to β-alanine metabolism. β-alanine is one of constituents of carnosine (β-alanyl-l-histidine) and anserine (β-alanyl-1-methyl-l-histidine), which are known to be present at high concentrations in the breast muscle of chickens and to exert antioxidant activity [35]. In this study, we found that the chickens fed the basal diet and kept at a HT had the lowest carnosine content in the breast muscle of the three treatment groups. This low carnosine content may explain the higher lipid peroxidation level in the breast muscle samples from this group.

An HT-induced decrease in the muscle carnosine content was also observed by Yang et al. [36], while Tomonaga et al. [15] reported that the constituents of carnosine (β-alanine and histidine) in chicks decreased in response to short-term chronic heat exposure. In this study, although the plasma dihydrouracil concentration was the highest, the plasma level of β-alanine was the lowest in the chickens fed the basal diet and kept under the HT conditions. Therefore, these results suggest that the conversion of dihydrouracil to β-alanine may be disrupted in chickens kept under HT conditions.

This study also evaluated the effects of feeding orotic acid to chickens, and found that it did not affect growth performance, as indicated by the final body weight, body weight gain, feed conversion ratio, feed intake, and tissue weights. However, orotic acid did ameliorate the HT-induced increases in lipid peroxidation level in the plasma of the chickens. This amelioration may have partially been due to the antioxidative activity of orotic acid [37], with the plasma level of orotic acid decreasing under the HT conditions and increasing in response to the orotic acid supplementation. The GC-MS/MS-based metabolomics analysis of the plasma indicated that 12 metabolites were affected by orotic acid supplementation, some of which have been reported to show antioxidative activity (e.g., uridine and 3-hydroxyanthranilic acid) [38,39] and appeared to be increased by orotic acid supplementation. Furthermore, the high-performance liquid chromatography analysis revealed that orotic acid significantly affected the plasma concentrations of aspartic acid, glutamic acid, and tyrosine. Of these three amino acids, aspartic acid and glutamic acid appeared to be increased by the orotic acid supplementation. Aspartic acid is converted into glutamic acid via the citric acid cycle [40], and glutamic acid is a component amino acid of glutathione, a well-known natural antioxidant [41,42]. These results suggest that alterations in the concentrations of these metabolites and amino acids may contribute to the amelioration of the HT environment-induced increase in the plasma lipid peroxidation level. Furthermore, orotic acid supplementation significantly affected the carnosine content of the breast muscle of the chickens. As previously mentioned, carnosine plays a role as an antioxidant [35], and the reduction of the lipid peroxidation level in the breast muscle tissue may therefore be linked to the orotic acid-induced increase in the carnosine content.

The GC-MS/MS-based metabolomics analysis indicated that orotic acid affected the plasma levels of pyrimidine-related metabolites (orotic acid, uracil, uridine, 2’-deoxyuridine, and β-alanine). This effect was likely linked to the entry of orotic acid into the de novo synthesis pathway for pyrimidines beyond the rate-limiting step. Furthermore, the de novo synthesis pathways for both pyrimidines and purines require 5-phosphoribosyl-1-pyrophosphate (PRPP), and the synthesis of PRPP by PRPP synthetase is the rate-limiting step for both pathways. In rats, it has been reported that orotic acid stimulates hepatic purine biosynthesis [43]. These results suggest that orotic acid may up-regulate the de novo synthesis of purines by providing additional PRPP. However, there was no significant interaction between the temperature and dietary treatments for the plasma metabolites. Further studies are required to understand the effects of orotic acid supplementation on the regulation of the de novo synthesis pathways for pyrimidines and purines in chickens.

The enrichment analysis identified changes in not only pyrimidine metabolism, but also in β-alanine metabolism, the malate–aspartate shuttle, and aspartate metabolism, in response to supplementing orotic acid. Of these, β-alanine metabolism and aspartate metabolism may be particularly important for the alleviation of HT-induced negative effects, as they were also found to be altered by HT conditions. Orotic acid supplementation was found to significantly increase the plasma level of β-alanine and the carnosine content of the breast muscles of the chickens. In chicks, orally administered β-alanine increased the carnosine content of the brain and muscles in a dose-dependent manner [44]. Therefore, these results suggest that orotic acid increases the muscle carnosine content by altering β-alanine metabolism, and thereby maintains the antioxidative capacity of broiler chickens under HT conditions. However, the mechanisms by which orotic acid supplementation increased the plasma level of β-alanine, even under HT conditions, remain unclear. Further research evaluating the effects of HT and orotic acid supplementation on the gene expression and activities of enzymes related to the conversion of dihydrouracil to β-alanine (i.e., dihydropyrimidinase and β-ureidopropionase) in chickens is necessary if we are to improve our insight into the reason for the HT-induced decrease in the muscle carnosine content and the alleviating effects of orotic acid supplementation.

As mentioned above, HT conditions severely increase lipid peroxidation levels [6,7], and consequently reduce meat quality (i.e., increase in drip loss, decrease in share force value, and change in meat color) [8,9,10,11,12,13,14]. The breast muscles of the chickens kept under cyclic HT conditions showed the highest lipid peroxidation levels, in conjunction with the lowest carnosine content, suggesting that their meat was of lower quality than that of the control chickens. In contrast, orotic acid increased the muscle carnosine content, possibly via pyrimidine metabolism and β-alanine metabolism, and consequently alleviated the HT environment-induced increase in the muscle lipid peroxidation levels. Cong et al. [45] reported that dietary supplementation of carnosine improved meat quality, antioxidant capacity, and lipid peroxidation status in broiler chickens. In addition, carnosine exerts antioxidant activity, even in cocked chicken meat [35]. These results suggest that orotic acid supplementation may have a positive effect on either carnosine content in meat or the meat quality of chickens.

## 4. Materials and Methods

### 4.1. Animals and Experimental Design

All experimental protocols and procedures were reviewed and approved by the Animal Care and Use Committee of Kagoshima University (approval number A18010). One hundred 1-day-old male broiler chicks (Chunky strain ROSS 308) were obtained from a commercial hatchery (Kumiai Hina Center, Kagoshima, Japan). Chicks were housed in an electrically-heated battery brooder and provided with water and a commercial diet (23% crude protein, 12.8 MJ/kg; Nichiwa Sangyou Company, Hyogo, Japan) until they were 14 days old. On day 14, 32 chicks were randomly selected from the group of 100. These chicks were housed individually in wire-bottomed aluminum cages (50 × 40 × 60 cm) and fed the basal diet (Table 7) for 3 days until the start of the main experimental period. The chicks were then randomly allocated to one of four groups, with the main experimental factors being diet and ambient temperature, in a 2 × 2 factorial design. The dietary treatments consisted of the basal diet or the basal diet supplemented with 0.7% of Lactoserum (Matsumoto Trading Co., Ltd., Tokyo, Japan), a food grade orotic acid that contains more than 98% of orotic acid as monohydrate form (i.e., more than 87.9% of orotic acid), and the temperature treatments consisted of either a thermoneutral environment at 25 ± 1 °C or an HT environment at 35 ± 1 °C. The experiment was conducted in a temperature-controlled room with 24 h of light and 50–70% relative humidity. The chicks assigned to the HT treatment groups were kept at 35 ± 1 °C for 8 h every day to mimic a realistic summer environment. At 32 days old, all the chickens were weighed, anesthetized by carbon dioxide, and killed by cervical dislocation. The chickens were then dissected, and the weights of the breast muscles (pectoralis major muscle), breast tender muscles (pectoralis minor muscle), leg muscles (thigh and drumstick), livers, hearts, and abdominal fat tissue depots were recorded. Blood samples were collected in heparinized test tubes, centrifuged at 5900× *g* for 10 min at 4 °C to separate the plasma, and stored at −30 °C until analysis.

### 4.2. Determination of MDA Concentration

To evaluate the oxidative stress levels, the MDA concentrations in the breast muscles and plasma were determined colorimetrically, using the thiobarbituric acid reactive substances assay, as described by Yagi [46] and Ohkawa et al. [47].

### 4.3. Determination of Free Amino Acid Concentrations

The analysis of the free amino acid and carnosine concentrations in the plasma and breast muscle tissue samples from the chickens was performed using a pre-column technique with liquid chromatography, according to previously reported methods [48]. A liquid chromatography system with automated pre-column derivatization functionality was used in the analysis (Nexera X2; Shimadzu Corporation, Kyoto, Japan). A total of 21 compounds were measured in the analysis, including the following basic amino acids and associated molecules: alanine, anserine, arginine, asparagine, aspartic acid, carnosine, glutamic acid, glutamine, glycine, histidine, isoleucine, leucine, lysine, methionine, phenylalanine, proline, serine, threonine, tryptophan, tyrosine, and valine. The concentrations of the amino acids in the plasma are expressed in µmol/L, and those in the breast muscle are expressed in mg/100 g. In this study, alanine and anserine were not separated, and carnosine was not detected in the plasma.

### 4.4. Sample Preparation for GC-MS/MS Analysis

Fifty μL aliquots of plasma were suspended in 250 µL of methanol/chloroform/water (5:2:2), with 5 µL of 1 mg/mL 2-isopropylmalic acid as the internal standard. The samples were then mixed in a shaker at 1200 rpm at 37 °C for 30 min, and then centrifuged at 16,000× *g* at 4 °C for 5 min. Next, 225 µL of the supernatant was mixed with 200 µL of distilled water and vortex-mixed, followed by centrifugation at 16,000× *g* at 4 °C for 5 min. Subsequently, 250 µL of the supernatant was dried under a vacuum using a centrifugal evaporator (RD-400; Yamato Scientific, Tokyo, Japan), after cooling at −80 °C for 10 min. Methoxyamine hydrochloride in pyridine (20 mg/mL, 40 µL) was then added to the tubes, and they were vortex-mixed, then shaken at 1200× *g* at 30 °C for 90 min in the dark to allow oximation. N-methyl-N-trimethylsilyltrifluoroacetamine (20 µL) was then added to each tube, and the contents were vortex-mixed. To prepare trimethylsilyl derivatives, the tubes were shaken at 1200× *g* at 37 °C for 45 min in the dark.

### 4.5. GC-MS/MS Analysis and Data Processing

GC-MS/MS analysis was performed as previously described [49], using a GCMS-TQ8050 (Shimadzu Corporation, Kyoto, Japan). A 30 m × 0.25 mm (internal diameter) BPX-5 column (SGE, Melbourne, Australia) with a 0.25 µm film thickness was used, according to the method described in the Smart Metabolites Database (Shimadzu, Kyoto, Japan).

Data processing was performed using the Smart Metabolites Database (Shimadzu, Kyoto, Japan), MS-DIAL version 3.08 [50], and the MRMPROBS program version 2.42 [51]. Peaks were recorded for the 45−600 m/z mass range, and were automatically detected via MS-DIAL using the peak detection option of a minimum peak height of 2000. A data quality check was conducted using the thresholds of −10 < RI < 10, dot production > 0.8, and presence > 0.6, and the remaining data was then manually checked. Ultimately, 172 metabolites were identified in the plasma samples. The relative quantities of the metabolites were calculated using the peak areas of each metabolite relative to that of the internal standard (2-isopropylmalic acid), and expressed as a percentage of an arbitrary control set to 100%.

### 4.6. Statistical Analysis

The data were analyzed using two-way ANOVA, with individual comparisons being made using Tukey’s multiple comparison test. All analyses were performed using R [52]. Statistical significance was set at *p* < 0.05, and the data are expressed as the means ± standard error of the mean (SEM).

Quantitative enrichment analysis using the pathway-associated metabolite sets included in MetaboAnalyst 4.0 [53], which is an established tool for metabolite set enrichment analysis, was performed using the plasma metabolite compounds that were significantly affected by either the HT treatment or orotic acid supplementation. Differences were considered significant at *p* < 0.05.

## 5. Conclusions

A cyclic HT environment altered nucleic acid metabolism and increased lipid peroxidation levels in the plasma and breast muscle of broiler chickens. Feeding the pyrimidine precursor orotic acid to the chickens altered the plasma concentrations of metabolites mainly related to pyrimidine metabolism and β-alanine metabolism, and ameliorated the increases in lipid peroxidation levels caused by rearing the broiler chickens under HT conditions.

## Figures and Tables

**Table 1:** Effects of a cyclic high ambient temperature and feeding orotic acid on the growth performance parameters of broiler chickens.

	Thermoneutral Temperature (25 ± 1 °C)		High Ambient Temperature (35 ± 1 °C for 8 h/day)		T	O	T × O
	Control	Orotic Acid	Control	Orotic Acid			
Final body weight (g)	1177.96 ± 64.68	1175.04 ± 70.06	1085.43 ± 33.39	1140.79 ± 40.13	N.S.	N.S.	N.S.
Body weight gain (g)	786.36 ± 64.27	777.49 ± 68.44	691.74 ± 36.24	746.31 ± 35.78	N.S.	N.S.	N.S.
Feed intake (g)	1216.59 ± 74.66	1268.61 ± 101.63	1022.19 ± 65.40	1118.18 ± 64.43	<0.05	N.S.	N.S.
Feed conversion ratio	1.56 ± 0.04	1.64 ± 0.05	1.48 ± 0.06	1.50 ± 0.05	N.S.	N.S.	N.S.
Body temperature (°C)	40.18 ± 0.07 ^c^	40.68 ± 0.16 ^bc^	41.37 ± 0.20 ^a^	41.13 ± 0.15 ^ab^	<0.001	N.S.	<0.05

Results are expressed as mean ± standard error of the mean (SEM) (*n* = 8). Means with the same superscript letter within rows are not significantly different at *p* < 0.05. T: the effect of temperature; O: the effect of feeding orotic acid; T × O: the statistical interaction between temperature and feeding orotic acid; N.S.: not significant.

**Table 2:** Effects of a cyclic high ambient temperature and feeding orotic acid on the weights of tissues of broiler chickens (g).

	Thermoneutral Temperature (25 ± 1 °C)		High Ambient Temperature (35 ± 1 °C for 8 h/day)		T	O	T × O
	Control	Otrotic Acid	Control	Orotic Acid			
Breast muscle	207.91 ± 11.01 ^ab^	222.19 ± 13.22 ^a^	182.65 ± 6.23 ^b^	188.28 ± 4.70 ^ab^	<0.01	N.S.	N.S.
Breast tender muscle	45.83 ± 2.54 ^a^	45.98 ± 2.49 ^a^	40.25 ± 1.21 ^a^	41.52 ± 1.30 ^a^	<0.05	N.S.	N.S.
Leg muscles	226.10 ± 16.26 ^a^	215.90 ± 13.47 ^a^	216.70 ± 6.56 ^a^	220.00 ± 8.56 ^a^	N.S.	N.S.	N.S.
Liver	22.20 ± 1.97 ^ab^	23.41 ± 1.72 ^a^	17.78 ± 0.65 ^b^	19.35 ± 1.31 ^ab^	<0.05	N.S.	N.S.
Heart	6.31 ± 0.54 ^a^	6.44 ± 0.66 ^a^	3.83 ± 0.16 ^b^	4.38 ± 0.34 ^b^	<0.001	N.S.	N.S.
Abdominal fat tissue	3.88 ± 1.25 ^b^	4.35 ± 1.00 ^b^	9.71 ± 1.55 ^a^	9.83 ± 1.24 ^a^	<0.001	N.S.	N.S.

Results are expressed as mean ± standard error of the mean (SEM) (*n* = 8). Means with the same superscript letter within rows are not significantly different at *p* < 0.05. T: the effect of temperature; O: the effect of feeding orotic acid; T × O: the statistical interaction between temperature and feeding orotic acid; N.S.: not significant.

**Table 3:** Effects of a cyclic high ambient temperature and feeding orotic acid on plasma-free amino acids of broiler chickens (μM).

	Thermoneutral Temperature (25 ± 1 °C)		High Ambient Temperature (35 ± 1 °C for 8 h/day)		T	O	T × O
	Control	Orotic Acid	Control	Orotic Acid			
Aspartic acid	15.82 ± 1.63	19.18 ± 3.13	12.00 ± 0.70	15.72 ± 2.10	N.S.	0.04	N.S.
Glutamic acid	28.52 ± 6.09	36.27 ± 3.15	32.13 ± 1.92	38.20 ± 3.82	N.S.	0.02	N.S.
Asparagine	17.56 ± 2.33	12.83 ± 3.05	14.04 ± 1.60	13.33 ± 2.06	N.S.	N.S.	N.S.
Serine	131.48 ± 8.88 ^ab^	151.03 ± 11.88 ^a^	98.78 ± 10.14 ^b^	95.74 ± 6.92 ^b^	<0.001	N.S.	N.S.
Glutamine	134.46 ± 8.69 ^a^	116.84 ± 13.86 ^a^	85.65 ± 10.75 ^b^	81.62 ± 8.81 ^b^	<0.05	N.S.	N.S.
Histidine	124.47 ± 11.15	151.57 ± 11.35	155.82 ± 12.55	161.53 ± 17.13	N.S.	N.S.	N.S.
Glycine	65.28 ± 6.59	85.55 ± 9.03	73.16 ± 7.27	69.77 ± 6.17	N.S.	N.S.	N.S.
Threonine	11.04 ± 1.05	17.10 ± 3.01	12.36 ± 1.93	14.60 ± 3.79	N.S.	N.S.	N.S.
Arginine	57.92 ± 5.42	58.30 ± 10.47	73.64 ± 8.20	60.60 ± 7.94	N.S.	N.S.	N.S.
Tyrosine	30.25 ± 2.00 ^a^	22.66 ± 2.78 ^ab^	24.09 ± 4.25 ^ab^	15.67 ± 1.73 ^b^	<0.05	<0.01	N.S.
Valine	21.28 ± 2.46	24.30 ± 2.38	20.02 ± 2.05	18.24 ± 1.49	N.S.	N.S.	N.S.
Methionine	13.89 ± 1.11	13.49 ± 2.35	12.27 ± 1.49	12.00 ± 1.12	N.S.	N.S.	N.S.
Tryptophan	19.69 ± 0.99	19.19 ± 1.17	19.77 ± 1.39	18.31 ± 1.01	N.S.	N.S.	N.S.
Phenylalanine	32.98 ± 3.11	33.37 ± 4.31	31.49 ± 3.99	27.36 ± 1.36	N.S.	N.S.	N.S.
Isoleucine	44.95 ± 4.43	44.59 ± 5.31	40.63 ± 5.29	35.77 ± 1.26	N.S.	N.S.	N.S.
Leucine	12.57 ± 1.20	12.83 ± 1.47	11.59 ± 1.87	9.68 ± 0.88	N.S.	N.S.	N.S.
Lysine	59.65 ± 10.69	75.17 ± 13.33	83.31 ± 14.47	72.88 ± 12.45	N.S.	N.S.	N.S.
Proline	51.70 ± 5.46	55.55 ± 4.10	64.98 ± 7.26	64.56 ± 6.29	N.S.	N.S.	N.S.

Results are expressed as mean ± standard error of the mean (SEM) (*n* = 8). Means with the same superscript letter within rows are not significantly different at *p* < 0.05. T: the effect of temperature; O: the effect of feeding orotic acid; T × O: the statistical interaction between temperature and feeding orotic acid; N.S.: not significant.

**Table 4:** Effects of a cyclic high ambient temperature and feeding orotic acid on plasma metabolites of broiler chickens.

	Thermoneutral Temperature (25 ± 1 °C)		High Ambient Temperature (35 ± 1 °C for 8 h/day)		T	O	T × O
	Control	Orotic Acid	Control	Orotic Acid			
**Metabolites affected by temperature**							
Nicotinic acid	100 ± 40 ^ab^	76 ± 26 ^b^	248 ± 95 ^ab^	336 ± 94 ^a^	<0.01	N.S.	N.S.
Methionine	100 ± 25 ^ab^	136 ± 27 ^a^	39 ± 7 ^b^	68 ± 17 ^ab^	<0.01	N.S.	N.S.
Galactosamine	100 ± 25 ^a^	92 ± 28 ^a^	30 ± 10 ^ab^	20 ± 7 ^b^	<0.01	N.S.	N.S.
Uric acid	100 ± 28 ^b^	148 ± 55 ^ab^	521 ± 161 ^ab^	621 ± 201 ^a^	<0.01	N.S.	N.S.
Xanthine	100 ± 23 ^b^	269 ± 84 ^ab^	585 ± 176 ^a^	635 ± 112 ^a^	<0.01	N.S.	N.S.
Xanthosine monophosphate	100 ± 18	103 ± 26	61 ± 15	48 ± 11	<0.05	N.S.	N.S.
Oleic acid	100 ± 29	70 ± 22	36 ± 13	29 ± 9	<0.05	N.S.	N.S.
Thymine	100 ± 21	94 ± 18	155 ± 24	199 ± 55	<0.05	N.S.	N.S.
Aspartic acid	100 ± 30	149 ± 40	49 ± 11	62 ± 16	<0.05	N.S.	N.S.
Dihydrouracil	100 ± 21	119 ± 27	193 ± 48	189 ± 42	<0.05	N.S.	N.S.
Ascorbic acid	100 ± 16	103 ± 18	64 ± 11	72 ± 13	<0.05	N.S.	N.S.
Inosine	100 ± 22	100 ± 18	191 ± 62	192 ± 49	<0.05	N.S.	N.S.
Ornithine	100 ± 26	112 ± 22	62 ± 11	59 ± 16	<0.05	N.S.	N.S.
3-Phenyllactic acid	100 ± 17	114 ± 26	192 ± 65	257 ± 82	<0.05	N.S.	N.S.
Cysteine	100 ± 21	124 ± 25	60 ± 12	79 ± 12	<0.05	N.S.	N.S.
Glutaric acid	100 ± 25	135 ± 32	164 ± 44	258 ± 70	<0.05	N.S.	N.S.
2-Hydroxyglutaric acid	100 ± 23	105 ± 17	169 ± 47	165 ± 33	<0.05	N.S.	N.S.
Sucrose	100 ± 45	152 ± 70	35 ± 7	48 ± 11	<0.05	N.S.	N.S.
Asparagine	100 ± 23	108 ± 27	56 ± 10	72 ± 12	<0.05	N.S.	N.S.
Serine	100 ± 16	110 ± 17	77 ± 15	68 ± 14	<0.05	N.S.	N.S.
Hypoxanthine	100 ± 18	123 ± 22	145 ± 34	182 ± 30	<0.05	N.S.	N.S.
**Metabolites affected by orotic acid**							
Niacinamide	100 ± 17 ^a^	66 ± 12 ^ab^	95 ± 19 ^ab^	42 ± 10 ^b^	N.S.	<0.01	N.S.
β-Alanine	100 ± 25 ^ab^	191 ± 33 ^a^	78 ± 18 ^b^	164 ± 38 ^ab^	N.S.	<0.01	N.S.
Uridine	100 ± 16	167 ± 28	90 ± 19	171 ± 36	N.S.	<0.05	N.S.
Guanine	100 ± 18	147 ± 21	110 ± 19	156 ± 22	N.S.	<0.05	N.S.
3-Hydroxyanthranilic acid	100 ± 27 ^b^	166 ± 46 ^ab^	150 ± 22 ^ab^	271 ± 65 ^a^	N.S.	<0.05	N.S.
Glycerol 3-phosphate	100 ± 28	151 ± 44	66 ± 14	140 ± 17	N.S.	<0.05	N.S.
2’-Deoxyuridine	100 ± 15	152 ± 27	107 ± 30	171 ± 36	N.S.	<0.05	N.S.
Cytosine	100 ± 23 ^ab^	115 ± 38 ^ab^	74 ± 13 ^b^	195 ± 42 ^a^	N.S.	<0.05	N.S.
3-Aminoisobutyric acid	100 ± 34	189 ± 61	100 ± 16	269 ± 94	N.S.	<0.05	N.S.
Phenylacetic acid	100 ± 29	180 ± 55	59 ± 9	127 ± 35	N.S.	<0.05	N.S.
**Metabolites affected by temperature and orotic acid**							
Uracil	100 ± 18 ^c^	273 ± 55 ^ab^	173 ± 31 ^bc^	379 ± 67 ^a^	<0.05	<0.01	N.S.
Orotic acid	100 ± 45 ^ab^	221 ± 30 ^a^	28 ± 6 ^b^	160 ± 34 ^a^	<0.05	<0.01	N.S.

The relative quantities of the metabolites were means ± SEM (*n* = 8) and expressed as percentage of an arbitrary control set to 100%. Means with the same superscript letter within rows are not significantly different at *p* < 0.05. T: the effect of temperature; O: the effect of feeding orotic acid; T × O: the statistical interaction between temperature and feeding orotic acid; N.S.: not significant.

**Table 5:** Effects of a cyclic high ambient temperature and feeding orotic acid on metabolisms of broiler chickens.

Metabolism Name	*p*-Value
**Metabolic pathways affected by high ambient temperature**	
Purine metabolism	<0.01
Ammonia recycling	<0.01
Pyrimidine metabolism	<0.01
Homocysteine degradation	<0.05
Glutamate metabolism	<0.05
Urea cycle	<0.05
β-Alanine metabolism	<0.05
Glycine and serine metabolism	<0.05
Aspartate metabolism	<0.05
**Metabolic pathway affected by orotic acid**	
Pyrimidine metabolism	<0.01
β-Alanine metabolism	<0.01
Malate–aspartate shuttle	<0.01
Aspartate metabolism	<0.05

**Table 6:** Effects of a cyclic high ambient temperature and feeding orotic acid on free amino acids and carnosine in the breast muscle of broiler chickens (mg/100 g).

	Thermoneutral Temperature (25 ± 1 °C)		High Ambient Temperature (35 ± 1 °C for 8 h/day)		T	O	T × O
	Control	Orotic Acid	Control	Orotic Acid			
Aspartic acid	5.91 ± 0.78	6.99 ± 0.48	4.99 ± 0.32	5.93 ± 0.73	N.S.	N.S.	N.S.
Glutamic acid	18.74 ± 3.10	19.30 ± 2.69	21.29 ± 1.39	21.91 ± 1.48	N.S.	N.S.	N.S.
Asparagine	5.98 ± 0.56	4.91 ± 0.21	5.25 ± 0.25	5.41 ± 0.26	N.S.	N.S.	N.S.
Serine	21.37 ± 1.60 ^ab^	22.27 ± 1.59 ^a^	16.49 ± 1.41 ^b^	16.76 ± 1.45 ^b^	<0.05	N.S.	N.S.
Glutamine	25.59 ± 1.47 ^a^	21.98 ± 2.18 ^ab^	17.00 ± 1.50 ^b^	18.84 ± 2.23 ^b^	<0.05	N.S.	N.S.
Histidine	1.11 ± 0.32	0.57 ± 0.08	1.31 ± 0.27	0.79 ± 0.05	N.S.	<0.05	N.S.
Glycine	33.19 ± 11.45	31.47 ± 4.48	37.68 ± 7.35	35.29 ± 4.71	N.S.	N.S.	N.S.
Threonine	12.37 ± 1.82	15.21 ± 1.66	14.92 ± 0.53	13.97 ± 0.91	N.S.	N.S.	N.S.
Arginine	9.77 ± 1.25 ^b^	11.83 ± 1.34 ^ab^	16.63 ± 2.13 ^ab^	13.82 ± 1.34 ^ab^	<0.05	N.S.	N.S.
Tyrosine	5.65 ± 0.94	5.82 ± 0.47	6.74 ± 0.32	4.78 ± 0.24	N.S.	N.S.	N.S.
Valine	3.23 ± 0.35	4.19 ± 0.62	4.21 ± 0.28	3.82 ± 0.31	N.S.	N.S.	N.S.
Methionine	1.25 ± 0.27 ^b^	2.19 ± 0.42 ^a^	1.57 ± 0.13 ^b^	1.28 ± 0.25 ^b^	<0.05	<0.05	<0.05
Tryptophan	5.03 ± 0.27	5.62 ± 0.93	4.98 ± 0.41	4.74 ± 0.64	N.S.	N.S.	N.S.
Phenylalanine	3.60 ± 0.20	3.89 ± 0.38	4.62 ± 0.29	4.24 ± 0.28	<0.05	N.S.	N.S.
Isoleucine	2.66 ± 0.22	3.11 ± 0.50	2.76 ± 0.24	2.59 ± 0.26	N.S.	N.S.	N.S.
Leucine	4.03 ± 0.44	4.35 ± 0.59	4.02 ± 0.31	3.85 ± 0.34	N.S.	N.S.	N.S.
Lysine	6.30 ± 2.20	11.36 ± 1.56	11.44 ± 1.58	10.38 ± 1.01	N.S.	N.S.	N.S.
Proline	4.37 ± 0.53	4.40 ± 0.35	4.23 ± 0.27	4.71 ± 0.66	N.S.	N.S.	N.S.
Carnosine	364.01 ± 69.97 ^ab^	446.29 ± 41.85 ^a^	236.35 ± 26.80 ^b^	437.63 ± 34.67 ^ab^	N.S.	<0.05	N.S.

Results are expressed as mean ± standard error of the mean (SEM) (*n* = 8). Means with the same superscript letter within rows are not significantly different at *p* < 0.05. T: the effect of temperature; O: the effect of feeding orotic acid; T × O: the statistical interaction between temperature and feeding orotic acid; N.S.: not significant.

**Table 7:** Composition and analysis of the basal diet.

	Ingredients (g/100 g)
Corn meal	57.90
Soybean meal	34.00
Corn oil	4.30
CaCO_3_	0.66
CaHPO_4_	2.00
NaCl	0.50
DL-Methionine	0.14
Mineral and vitamin premix ^1^	0.50
**Calculated analysis**	
Crude protein (%)	20.00
Metabolizable energy (Mcal/kg)	3.10

^1^ Content per kg of the vitamin and mineral premix: vitamin A = 90 mg, vitamin D3 = 1 mg, DL-alpha-tocopherol acetate = 2000 mg, vitamin K3 = 229 mg, thiamin nitrate = 444 mg, riboflavin = 720 mg, calcium d-pantothenate = 2174 mg, nicotinamide = 7000 mg, pyridoxine hydrochloride = 700 mg, biotin = 30 mg, folic acid = 110 mg, cyanocobalamine = 2 mg, calcium iodinate = 108 mg, MgO = 198,991 mg, MnSO_4_ = 32,985 mg, ZnSO_4_ = 19,753 mg, FeSO_4_ = 43,523 mg, CuSO_4_ = 4019 mg, and choline chloride = 299,608 mg.

## Supplementary Materials

The following is available online at https://www.mdpi.com/2218-1989/10/5/189/s1, Table S1. Effects of a cyclic high ambient temperature and feeding orotic acid on the muscle yields of broiler chickens. Table S2. Metabolites identified in the plasma of broiler chickens reared under either thermoneutral or high ambient temperature (HT) conditions, and fed either a control or orotic acid-supplemented diet.
