# Supplementary material for: Effects of Cyclic High Ambient Temperature and Dietary Supplementation of Orotic Acid, a Pyrimidine Precursor, on Plasma and Muscle Metabolites in Broiler Chickens
Source: Metabolites. 2020 May 12;10(5):189. doi: 10.3390/metabo10050189 (PMC7281580; doi:10.3390/metabo10050189)
Supplement: Supplementary file 1 [file metabolites-10-00189-s001.pdf]

Table S1. Effects of a cyclic high ambient temperature and feeding orotic acid on the muscle yields of broiler chickens (g / 100g body weight).

	Thermoneutral temperature (25 ± 1 °C)		High ambient temperature (35 ± 1 °C for 8h/day)		T	O	T × O
	Control	Orotic acid	Control	Orotic acid			
Breast muscle	17.34 ± 0.37 ^{b}	18.74 ± 0.27 ^{a}	16.89 ± 0.35 ^{b}	16.96 ± 0.28 ^{b}	< 0.01	< 0.05	N.S.
Breast tender muscle	3.83 ± 0.15	3.81 ± 0.07	3.79 ± 0.07	3.77 ± 0.08	N.S.	N.S.	N.S.
Leg muscles	19.03 ± 0.41	18.58 ± 0.43	19.91 ± 0.42	19.22 ± 0.24	N.S.	N.S.	N.S.

Results are expressed as mean ± standard error of the mean (SEM) (n = 8). Means with the same superscript letter within rows are not significantly different at $P < 0.05$. T, the effect of temperature; O, the effect of feeding orotic acid; T × O, the statistical interaction between temperature and feeding orotic acid. N.S.: not significant.

Table S2. Plasma metabolites broiler chickens identified in this study.

	Thermoneutral temperature (25 ± 1 °C)		Heat ambient temperature (35 ± 1 °C for 8h/day)	
	**Control**	**Orotic acid**	**Control**	**Orotic acid**
3-Methoxy-4-hydroxybenzoic acid	100 ± 24	101 ± 22	77 ± 23	68 ± 10
Vanillylamine	100 ± 22	81 ± 15	82 ± 19	88 ± 18
5-Aminolevulinic acid	100 ± 11	137 ± 42	137 ± 46	115 ± 40
3-Phosphoglyceric acid	100 ± 27	130 ± 38	62 ± 13	86 ± 14
3-Hydroxyanthranilic acid	100 ± 27	166 ± 46	150 ± 22	271 ± 65
Dihydroxyacetone	100 ± 27	94 ± 22	102 ± 32	104 ± 24
Juniperic acid	100 ± 24	100 ± 26	161 ± 78	128 ± 35
Xanthosine	100 ± 19	123 ± 29	144 ± 34	159 ± 27
Cholesterol	100 ± 24	111 ± 23	90 ± 22	112 ± 37
Catechol	100 ± 18	78 ± 13	68 ± 16	103 ± 22
2-Aminooctanoic acid	100 ± 18	134 ± 31	85 ± 16	93 ± 19
4-Hydroxybenzoic acid	100 ± 18	115 ± 21	81 ± 27	100 ± 26
4-Hydroxyphenylacetic acid	100 ± 19	103 ± 22	82 ± 13	123 ± 32
Porphobilinogen	100 ± 23	113 ± 28	98 ± 26	97 ± 23
2-Hydroxyhippuric acid	100 ± 21	94 ± 22	77 ± 18	82 ± 20
N-Acetylaspartic acid	100 ± 20	115 ± 17	142 ± 34	143 ± 27
Methylsuccinic acid	100 ± 25	91 ± 21	78 ± 21	148 ± 64
Nicotinic acid	100 ± 40	76 ± 26	248 ± 95	336 ± 94
Fructose 6-phosphate	100 ± 22	136 ± 18	113 ± 29	139 ± 36
Sebacic acid	100 ± 18	112 ± 16	95 ± 21	104 ± 23
3-Aminopropanoic acid	100 ± 35	239 ± 68	111 ± 17	340 ± 115

Table S2. *Cont.*

	Thermoneutral temperature (25 ± 1 °C)		Heat ambient temperature (35 ± 1 °C for 8h/day)	
	**Control**	**Orotic acid**	**Control**	**Orotic acid**
Guanosine	100 ± 23	129 ± 20	129 ± 34	129 ± 28
Aconitic acid	100 ± 23	101 ± 31	53 ± 9	62 ± 6
Tartaric acid	100 ± 11	87 ± 9	102 ± 19	72 ± 5
O-Phosphoethanolamine	100 ± 19	157 ± 42	82 ± 19	133 ± 32
5-Hydroxymethyl-2-furoic acid	100 ± 6	102 ± 5	102 ± 5	95 ± 5
2-Aminoadipic acid	100 ± 21	121 ± 25	111 ± 31	113 ± 25
Urocanic acid	100 ± 19	118 ± 24	105 ± 22	136 ± 36
Dihydroxyacetone phosphate	100 ± 18	97 ± 20	87 ± 18	102 ± 21
N-Acetylserine	100 ± 15	108 ± 22	128 ± 30	128 ± 32
1-Hexadecanol	100 ± 25	110 ± 27	87 ± 13	93 ± 28
3-Dehydroshikimic acid	100 ± 18	97 ± 18	95 ± 20	101 ± 18
5-Hydroxy-tryptophan	100 ± 19	142 ± 27	87 ± 19	79 ± 20
Pyrogallol	100 ± 23	81 ± 18	83 ± 21	95 ± 21
Phenylacetic acid	100 ± 29	180 ± 55	59 ± 9	127 ± 35
Epinephrine	100 ± 19	75 ± 18	77 ± 19	65 ± 9
Quinolinic acid	100 ± 19	132 ± 24	114 ± 27	274 ± 145
Pyruvic acid	100 ± 28	95 ± 20	81 ± 16	74 ± 17
Pantothenic acid	100 ± 20	101 ± 16	68 ± 16	103 ± 24
Docosahexaenoic acid	100 ± 15	104 ± 19	82 ± 16	78 ± 15
2-Hydroxyisovaleric acid	100 ± 25	101 ± 21	145 ± 35	144 ± 44
Glutamine	100 ± 47	75 ± 21	36 ± 11	35 ± 17
2-Ketoadipic acid	100 ± 11	77 ± 14	131 ± 34	123 ± 33

Table S2. *Cont.*

	Thermoneutral temperature (25 ± 1 °C)		Heat ambient temperature (35 ± 1 °C for 8h/day)	
	**Control**	**Orotic acid**	**Control**	**Orotic acid**
Maleic acid	100 ± 16	103 ± 20	104 ± 24	115 ± 22
Erythrose 4-phosphate	100 ± 20	92 ± 17	90 ± 21	90 ± 19
Creatinine	100 ± 22	102 ± 15	144 ± 53	114 ± 20
Oxalacetic acid	100 ± 20	129 ± 23	91 ± 21	133 ± 30
Citramalic acid	100 ± 17	87 ± 15	121 ± 27	106 ± 24
Ribulose	100 ± 18	99 ± 15	113 ± 26	116 ± 22
Histamine	100 ± 18	100 ± 16	87 ± 20	92 ± 18
Palmitoleic acid	100 ± 26	61 ± 18	59 ± 23	37 ± 10
6-Phosphogluconic acid	100 ± 20	113 ± 30	68 ± 20	70 ± 19
2-Hydroxyglutaric acid	100 ± 23	105 ± 17	169 ± 47	165 ± 33
Lauric acid	100 ± 22	105 ± 21	98 ± 24	95 ± 26
Caproic acid	100 ± 27	127 ± 52	77 ± 18	104 ± 29
Adenine	100 ± 17	103 ± 17	92 ± 19	102 ± 20
3-Methyl-2-oxovaleric acid	100 ± 18	90 ± 17	98 ± 24	83 ± 18
O-Acetylserine	100 ± 19	125 ± 26	88 ± 17	91 ± 17
Margaric acid	100 ± 20	106 ± 22	88 ± 17	78 ± 18
2-Hydroxyisobutyric acid	100 ± 25	92 ± 13	72 ± 16	137 ± 40
Octadecanol	100 ± 20	107 ± 22	84 ± 17	76 ± 17
N-Acetyl-Ornithine	100 ± 16	121 ± 26	91 ± 22	101 ± 17
Glucose 6-phosphate	100 ± 16	137 ± 27	94 ± 19	107 ± 27
2-Aminoethanol	100 ± 17	84 ± 12	105 ± 22	110 ± 23
Niacinamide	100 ± 17	66 ± 12	95 ± 19	42 ± 10

Table S2. *Cont.*

	Thermoneutral temperature (25 ± 1 °C)		Heat ambient temperature (35 ± 1 °C for 8h/day)	
	**Control**	**Orotic acid**	**Control**	**Orotic acid**
Histidine	100 ± 27	128 ± 40	138 ± 37	120 ± 32
Rhamnose	100 ± 16	104 ± 18	101 ± 20	110 ± 24
Ribose 5-phosphate	100 ± 21	103 ± 22	85 ± 18	74 ± 18
Xylulose	100 ± 18	101 ± 14	114 ± 25	131 ± 22
Fucose	100 ± 16	94 ± 16	98 ± 20	102 ± 22
Isoleucine	100 ± 24	90 ± 21	99 ± 22	84 ± 19
2-Ketoisocaproic acid	100 ± 15	90 ± 17	94 ± 23	85 ± 15
Glycolic acid	100 ± 16	116 ± 18	121 ± 29	123 ± 25
Malic acid	100 ± 19	92 ± 16	101 ± 24	112 ± 28
Fumaric acid	100 ± 17	97 ± 15	98 ± 22	104 ± 23
Phenylpyruvic acid	100 ± 19	100 ± 18	91 ± 17	85 ± 18
2-Aminobutyric acid	100 ± 27	98 ± 19	81 ± 16	77 ± 19
Nonanoic acid	100 ± 16	106 ± 14	95 ± 13	92 ± 16
Ribonic acid	100 ± 17	110 ± 19	106 ± 21	123 ± 25
Glyceraldehyde	100 ± 19	106 ± 24	104 ± 25	97 ± 20
Linoleic acid	100 ± 30	70 ± 22	57 ± 28	31 ± 10
Hypotaurine	100 ± 15	92 ± 17	86 ± 19	85 ± 22
Threitol	100 ± 20	81 ± 16	109 ± 24	107 ± 23
Isocitric acid	100 ± 20	97 ± 17	100 ± 21	103 ± 22
2-Hydroxybutyric acid	100 ± 21	90 ± 17	164 ± 44	132 ± 32
Pyridoxamine	100 ± 22	92 ± 20	101 ± 25	61 ± 11
Glycine	100 ± 26	63 ± 14	138 ± 36	113 ± 33

Table S2. *Cont.*

	Thermoneutral temperature (25 ± 1 °C)						Heat ambient temperature (35 ± 1 °C for 8h/day)					
	Control			Orotic acid			Control			Orotic acid		
Benzoic acid	100	±	12	93	±	10	87	±	8	93	±	9
Eicosapentaenoic acid	100	±	21	91	±	20	73	±	18	54	±	14
Threonic acid	100	±	17	109	±	21	105	±	22	92	±	21
2-Ketoglutaric acid	100	±	15	115	±	20	134	±	33	153	±	42
Ureidosuccinic acid	100	±	15	109	±	20	75	±	15	87	±	15
1,6-Anhydroglucose	100	±	20	94	±	19	88	±	20	97	±	20
Octopamine	100	±	19	99	±	17	102	±	20	103	±	23
Succinic acid	100	±	19	105	±	23	94	±	21	102	±	25
Allantoin	100	±	17	103	±	18	85	±	18	93	±	20
Tryptamine	100	±	19	101	±	19	80	±	18	95	±	25
Sucrose	100	±	45	152	±	70	35	±	7	48	±	11
Galactosamine	100	±	25	92	±	28	30	±	10	20	±	7
Lysine	100	±	32	139	±	46	159	±	48	128	±	35
Glucosamine	100	±	17	97	±	16	85	±	17	89	±	19
Galacturonic acid	100	±	14	120	±	22	107	±	23	132	±	30
Glucuronic acid	100	±	14	120	±	22	107	±	23	132	±	30
Dimethylglycine	100	±	20	92	±	12	86	±	15	76	±	14
3-Hydroxyglutaric acid	100	±	1	101	±	0	101	±	1	100	±	1
N-Acetyl-Lysine	100	±	16	99	±	16	86	±	18	87	±	18
Tyramine	100	±	31	138	±	44	152	±	44	125	±	34
N-Acetylmannosamine	100	±	17	105	±	19	127	±	27	132	±	33
5'-Methylthioadenosine	100	±	17	110	±	18	96	±	19	96	±	22

Table S2. *Cont.*

	**Thermoneutral temperature (25 ± 1 °C)**		**Heat ambient temperature (35 ± 1 °C for 8h/day)**	
	**Control**	**Orotic acid**	**Control**	**Orotic acid**
2-Aminopimelic acid	100 ± 21	96 ± 18	100 ± 21	103 ± 22
Phenylalanine	100 ± 20	115 ± 24	79 ± 21	79 ± 17
Tryptophan	100 ± 21	121 ± 29	100 ± 21	84 ± 23
Glutamic acid	100 ± 21	124 ± 23	83 ± 18	91 ± 21
Citric acid	100 ± 21	95 ± 17	100 ± 21	104 ± 22
Tyrosine	100 ± 20	96 ± 19	71 ± 10	67 ± 12
3-Hydroxyisobutyric acid	100 ± 26	97 ± 20	102 ± 20	87 ± 24
Threonine	100 ± 20	126 ± 26	86 ± 16	88 ± 17
4-Hydroxyproline	100 ± 20	129 ± 23	92 ± 19	130 ± 27
Kynurenic acid	100 ± 14	110 ± 18	154 ± 35	145 ± 31
Stearic acid	100 ± 19	109 ± 21	87 ± 17	76 ± 18
Fructose	100 ± 21	87 ± 15	85 ± 19	87 ± 18
Valine	100 ± 20	97 ± 19	95 ± 23	90 ± 17
Leucine	100 ± 19	93 ± 16	79 ± 20	80 ± 14
Proline	100 ± 19	96 ± 15	98 ± 24	104 ± 20
Palmitic acid	100 ± 22	93 ± 18	84 ± 19	74 ± 17
5-Oxoproline	100 ± 18	91 ± 14	81 ± 18	75 ± 13
Inositol	100 ± 17	97 ± 15	111 ± 26	113 ± 22
Alanine	100 ± 23	81 ± 12	79 ± 18	83 ± 17
Monostearin	100 ± 22	88 ± 15	85 ± 19	85 ± 18
Lactic acid	100 ± 23	80 ± 12	86 ± 20	91 ± 18
Phosphoric acid	100 ± 21	83 ± 13	85 ± 20	87 ± 16

Table S2. *Cont.*

	**Thermoneutral temperature (25 ± 1 °C)**		**Heat ambient temperature (35 ± 1 °C for 8h/day)**	
	**Control**	**Orotic acid**	**Control**	**Orotic acid**
Galactose	100 ± 24	82 ± 13	86 ± 19	95 ± 16
Mannose	100 ± 24	82 ± 13	79 ± 18	87 ± 17
Glucose	100 ± 24	83 ± 13	79 ± 18	87 ± 17
Sorbitol	100 ± 24	83 ± 13	79 ± 18	87 ± 17
Mannitol	100 ± 25	82 ± 13	79 ± 19	86 ± 17
Allose	100 ± 20	100 ± 16	95 ± 22	105 ± 21
Glucono-1,5-lactone	100 ± 24	81 ± 13	79 ± 18	85 ± 17

The relative quantities of the metabolites were means ± SEM (n = 8) and expressed as percentage of an arbitrary control set to 100%.
